# Resilience and regime shifts in a marine biodiversity hotspot

**DOI:** 10.1038/s41598-017-13852-9

**Published:** 2017-10-20

**Authors:** Paraskevas Vasilakopoulos, Dionysios E. Raitsos, Evangelos Tzanatos, Christos D. Maravelias

**Affiliations:** 1 Institute of Marine Biological Resources and Inland Waters, Hellenic Centre for Marine Research (HCMR), 46.7 km Athens-Sounio ave., Anavyssos, 19013 Greece; 2 European Commission, Joint Research Centre, Directorate D - Sustainable Resources, Unit D.02 Water and Marine Resources, Via Enrico Fermi 2749, 21027 Ispra (VA), Italy; 3 Remote Sensing Group, Plymouth Marine Laboratory (PML), Plymouth, Devon PL1 3DH UK; 4 National Centre for Earth Observation, PML, Plymouth, UK; 5 Department of Biology, University of Patras, 26504 Patras, Greece

## Abstract

Complex natural systems, spanning from individuals and populations to ecosystems and social-ecological systems, often exhibit abrupt reorganizations in response to changing stressors, known as regime shifts or critical transitions. Theory suggests that such systems feature folded stability landscapes with fluctuating resilience, fold-bifurcations, and alternate basins of attraction. However, the implementation of such features to elucidate response mechanisms in an empirical context is scarce, due to the lack of generic approaches to quantify resilience dynamics in individual natural systems. Here, we introduce an Integrated Resilience Assessment (IRA) framework: a three-step analytical process to assess resilience and construct stability landscapes of empirical systems. The proposed framework involves a multivariate analysis to estimate holistic system indicator variables, non-additive modelling to estimate alternate attractors, and a quantitative resilience assessment to scale stability landscapes. We implement this framework to investigate the temporal development of the Mediterranean marine communities in response to sea warming during 1985–2013, using fisheries landings data. Our analysis revealed a nonlinear tropicalisation of the Mediterranean Sea, expressed as abrupt shifts to regimes dominated by thermophilic species. The approach exemplified here for the Mediterranean Sea, revealing previously unknown resilience dynamics driven by climate forcing, can elucidate resilience and shifts in other complex systems.

## Introduction

Complex natural systems are exposed to strong natural and anthropogenic stressors, which are currently affecting them at alarming rates^1,2^. In particular, climate change has been shown to affect the global biosphere by causing large-scale reorganisations in populations, communities and ecosystems^3–5^. An advanced understanding of how different natural systems reorganise themselves in the face of change is required to ensure environmental integrity, food security, and sustainable economic growth^3^.

Complex systems respond to changing stressors either in a continuous, or in a discontinuous way^3,6,7^. In a continuous response, a system changes linearly in relation to the corresponding change of its stressors, while in a discontinuous response the system response curve is folded backwards, forming a fold-bifurcation with two tipping points (Supplementary Fig. S1). A fold-bifurcation implies that a system crossing a tipping point will switch abruptly to a different regime corresponding to an alternate configuration, in what is termed a ‘regime shift’ or ‘critical transition’^3^. Regime shifts have been detected in multiple ecosystems via Integrated Ecosystem Assessments (IEAs), which may involve, among others, multivariate analysis and non-additive modelling^8–11^. Regime shifts have serious implications for the structure and function of natural systems, they are challenging to predict, and they are often irreversible due to the effect of hysteresis^3,9^.

Alternate regimes refer to dynamic states exhibiting a defined range of deviations from an attractor over time^12^; thus, regimes can be viewed as basins of attraction around the system response curves (attractors), forming a folded stability landscape (Supplementary Fig. S2a). In this context, ecological resilience (‘resilience’ hereafter) describes the capacity of complex natural systems with alternate attractors to persist within their original regime as conditions change^13^. Despite being an important concept, resilience has been mainly used as a theoretical construct with a qualitative interpretation^14–16^. In recent years, significant efforts have been made to develop methods for quantifying resilience in simulated, manipulated, or natural systems. Scheffer *et al*.^16^ identify two sets of such methods: the first set is based on quantifying indicators of critical slowing down, i.e. the slower recovery from small perturbations close to a tipping point, such as changes in the fluctuations or spatial patterns of a system^17–20^; the second set quantifies the resilience of alternate states in probabilistic terms using information from a large number of different systems^21,22^, or simulated system states^23–25^. These methods provide informative, albeit indirect, quantifications of resilience, but require large datasets with high temporal resolution which are usually unavailable for empirical natural systems. A third approach to quantify resilience and construct stability landscapes is the direct calculation of the distance of system states from a tipping point^26^, or from both a tipping point and an attractor^27^ (Supplementary Fig. S2b). This latter approach can be applied to individual systems where available empirical datasets do not allow for the quantification of critical slowing down indicators or the implementation of probabilistic approaches; however, it has not been tested in natural systems of high complexity such as communities or ecosystems. Quantifying resilience and folded stability landscapes of empirical complex systems in a simple and reproducible way could enhance our understanding of their non-linear dynamics, elucidate shift mechanisms, and allow the identification of stressor levels corresponding to tipping points.

**Figure 1:**
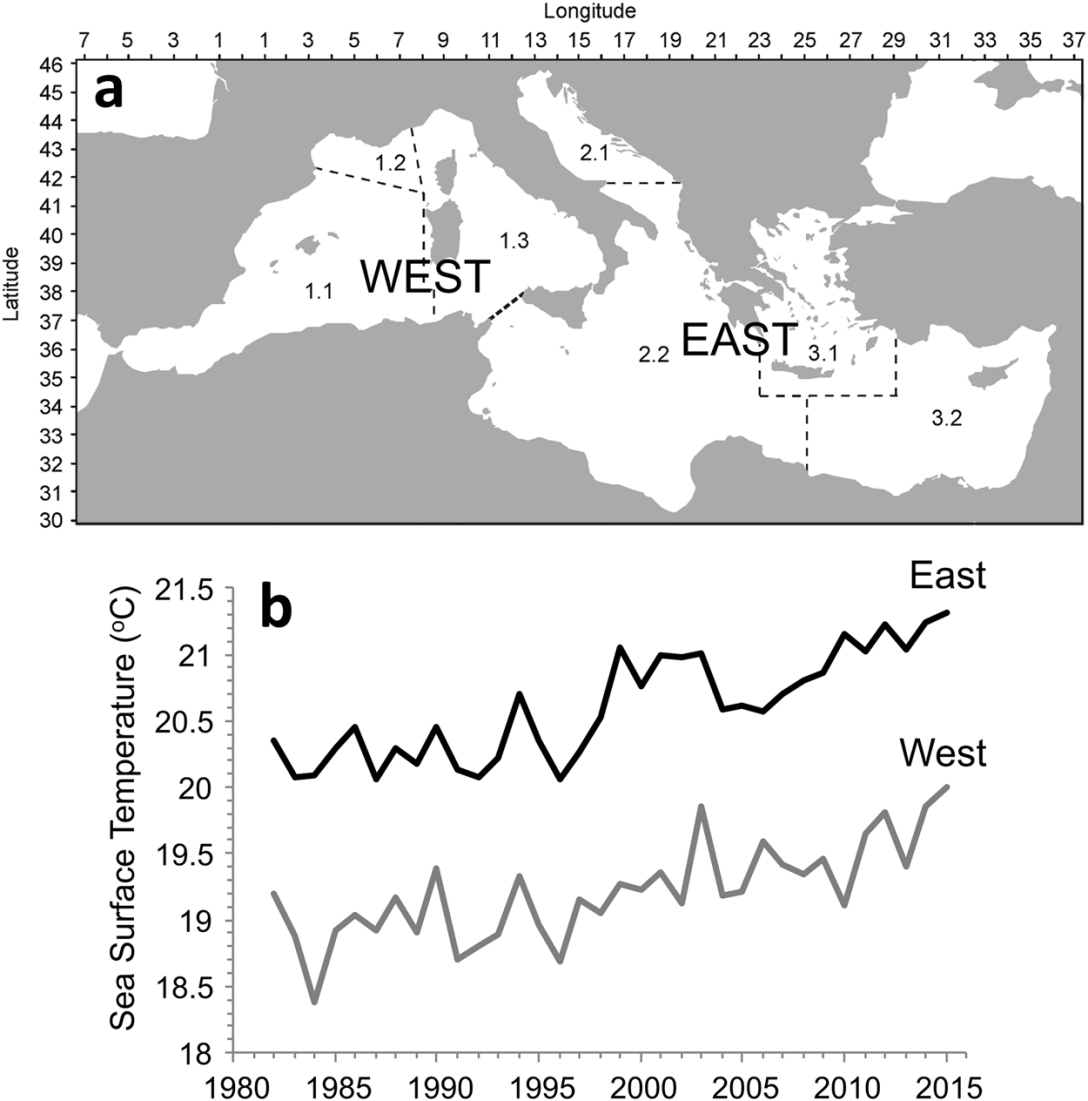
The studied areas of the Mediterranean Sea and the temporal development of SST in each one of them in 1982–2015. (**a**) The western Mediterranean included FAO areas 1.1 (Balearic), 1.2 (Gulf of Lions) and 1.3 (Sardinia), while the eastern Mediterranean included FAO areas 2.1 (Adriatic), 2.2 (Ionian), 3.1 (Aegean) and 2.2 (Levant) (created using R version 3.3.3; https://www.r-project.org/). (**b**) The temporal development of SST in the eastern Mediterranean (black line) and in the western Mediterranean (grey line).

Here, we combine multivariate and non-additive modelling tools^8,9^, and extend the resilience quantification introduced by Vasilakopoulos & Marshall^27^, to develop a three-step Integrated Resilience Assessment (IRA) framework (Table 1). We apply this framework to empirical biological data-series (fisheries landings) from the eastern and western Mediterranean Sea (Fig. 1a) during 1985-2013, and investigate the effect of the concurrent development of sea surface temperature (SST). The Mediterranean Sea is a biodiversity hotspot^28^, and due to its documented long-going human exploitation and climatic change, it serves as an excellent ‘laboratory’ for climate, environmental, and management studies^29^. While some large-scale changes in Mediterranean marine communities in response to sea warming have been previously documented^4,30–32^, it has not been ratified whether the Mediterranean fisheries resources have undergone regime shifts at an entire assemblage level. Additionally, the response type of the Mediterranean communities to sea warming, potential multivariate shifts, shift mechanisms, and resilience dynamics remain unknown. Fisheries landings time-series of 30 fish, crustacean and molluscan taxa (species or groups of species) were analysed here; hence, in this study, ‘regime’ refers to the configuration of the multivariate fisheries landings profile (‘system’ hereafter). Fisheries landings were extracted from the official capture production database of the Food and Agriculture Organisation (FAO) of the United Nations, provided by FAO GFCM (General Fisheries Commission for the Mediterranean). The analysis was carried out separately for the eastern and western Mediterranean basins, due to their different ecological structures^33^ and warming profiles^34^ (Fig. 1b). Our analysis elucidates the way that changes in SST led to resilience erosion and regime shifts in the Mediterranean marine communities, in a way that is transferable to any other complex natural system.

**Table 1:** The Integrated Resilience Assessment (IRA) framework.

**Step 1: Complexity reduction**. The complexity of the empirical system is reduced down to a single (or a few) system indicator variables. This process requires all individual variables describing the complex system to have the same spatial and temporal resolution^9^. By implementing a multivariate analysis on the time-variable matrix, such as a principal component analysis (PCA) or some other dimension reduction method, new composite variables (e.g. principal components) can be produced, capturing a high proportion of the information contained in the original variables. Such a multivariate analysis has the additional advantage of allowing the detection of system regime shifts expressed as shifts in the mean value of the indicator variable, albeit without clarifying whether these shifts are due to continuous or discontinuous responses to changing stressors. A multivariate analysis may not be necessary if an original system variable is considered representative of the multivariate complex system, in which case that variable can be used as system state indicator^22,23^.
**Step 2: Non-additive modelling**. One or more system indicator variables are linked to the concurrent development of one or more stressors of the system to reveal the response mechanism at play. If the aim is to examine the confounding effects of multiple stressors, a separate dimension reduction analysis (see Step 1) can be applied to the stressor variables in order to estimate a multivariate stressor indicator^27^. The fit of additive (continuous) statistical models, such as generalised additive models (GAMs), on the system-stressor relationship is then compared to the respective fit of non-additive (discontinuous) statistical models, such as threshold GAMs (TGAMs), to assess the type (continuous/discontinuous) of the system response^8,35^. Possible lagged stressor effects can also be investigated in this context. In the case of a discontinuous system response (i.e. TGAMs providing better fit than GAMs), the lines of the fitted models represent the alternate attractors. The position of the tipping points can then be approximated based on the thresholds identified by the non-additive models, and one or more fold-bifurcations can be revealed.
**Step 3: Resilience assessment**. The resilience of each observed system state within a fold-bifurcation is quantified based on the distance of that state from its attractor and tipping point^27^. If we define the horizontal component of resilience (*hComp*) to be the horizontal distance of a system state from the tipping point of its attractor curve, and the vertical component of resilience (*vComp*) to be the negative vertical distance of a system state from its attractor, resilience (*Res*) of a system at any point of time can be approximated as: *Res* = *vComp* + *hComp*, where by definition *vComp* ≤ 0 and *hComp* ≥ 0 (Supplementary Fig. S2b). This way, resilience estimates of all observed system-stressor combinations can be produced. This process requires both the system and stressor variables to be measured at the same scale; hence, a standardisation of the system and/or stressor indicator variables may be necessary prior to the resilience assessment. By setting the resilience of tipping points to zero and interpolating all resilience estimates across the stability landscape, the folded stability landscape with its alternate basins of attraction will emerge (Supplementary Fig. S2a).

## Results

### Multivariate analysis and complexity reduction

**Figure 2:**
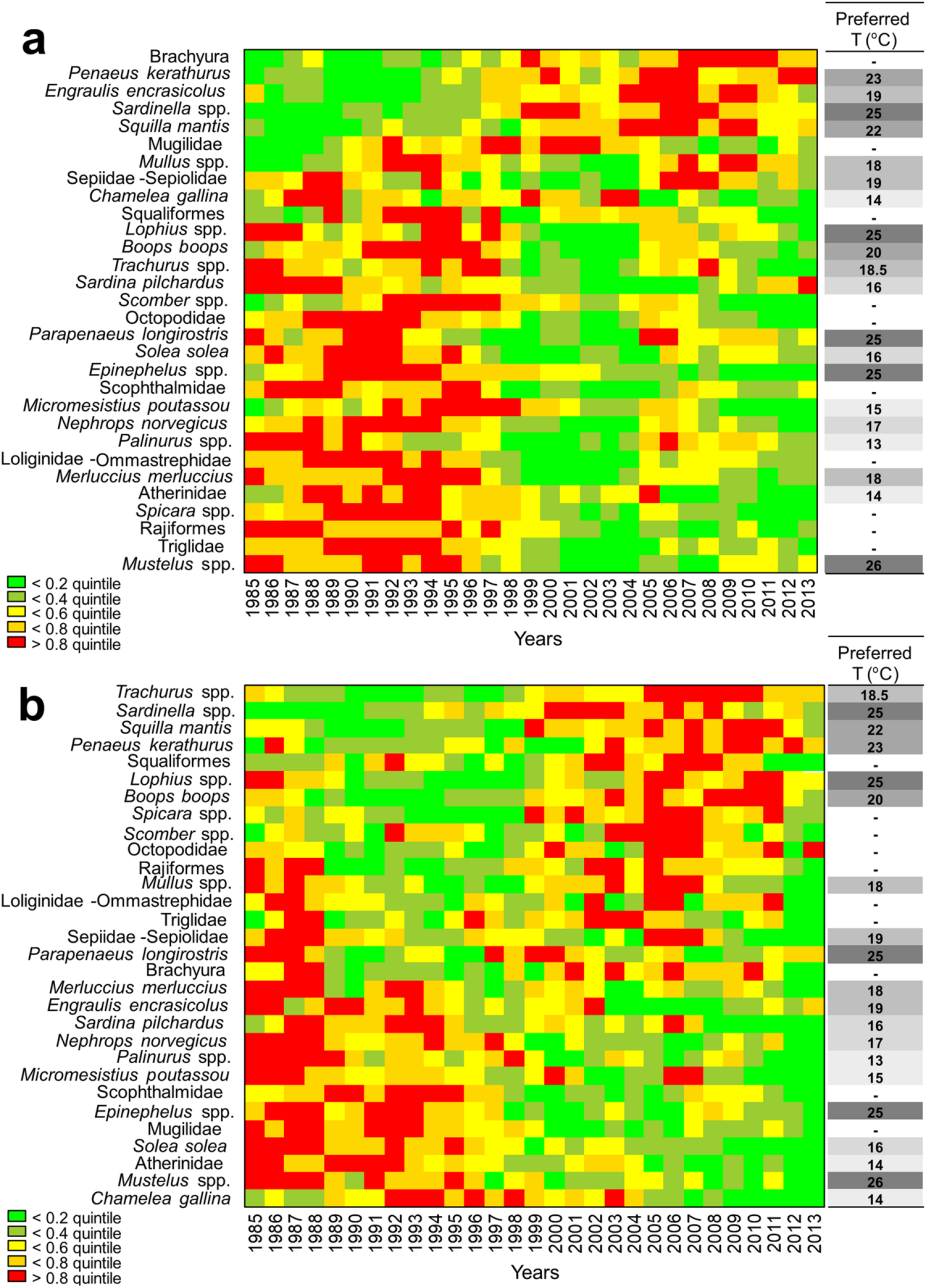
Traffic light plots for the 30 analysed taxa and their preferred temperatures. (**a**) Temporal development of landings and preferred temperatures of the eastern Mediterranean taxa; taxa have been sorted according to their loadings on PC1east. (**b**) Temporal development of landings and preferred temperatures of the western Mediterranean taxa; taxa have been sorted according to their loadings on PC1west. Five grayscale levels have been used for preferred temperatures indicating successive quintiles.

**Figure 3:**
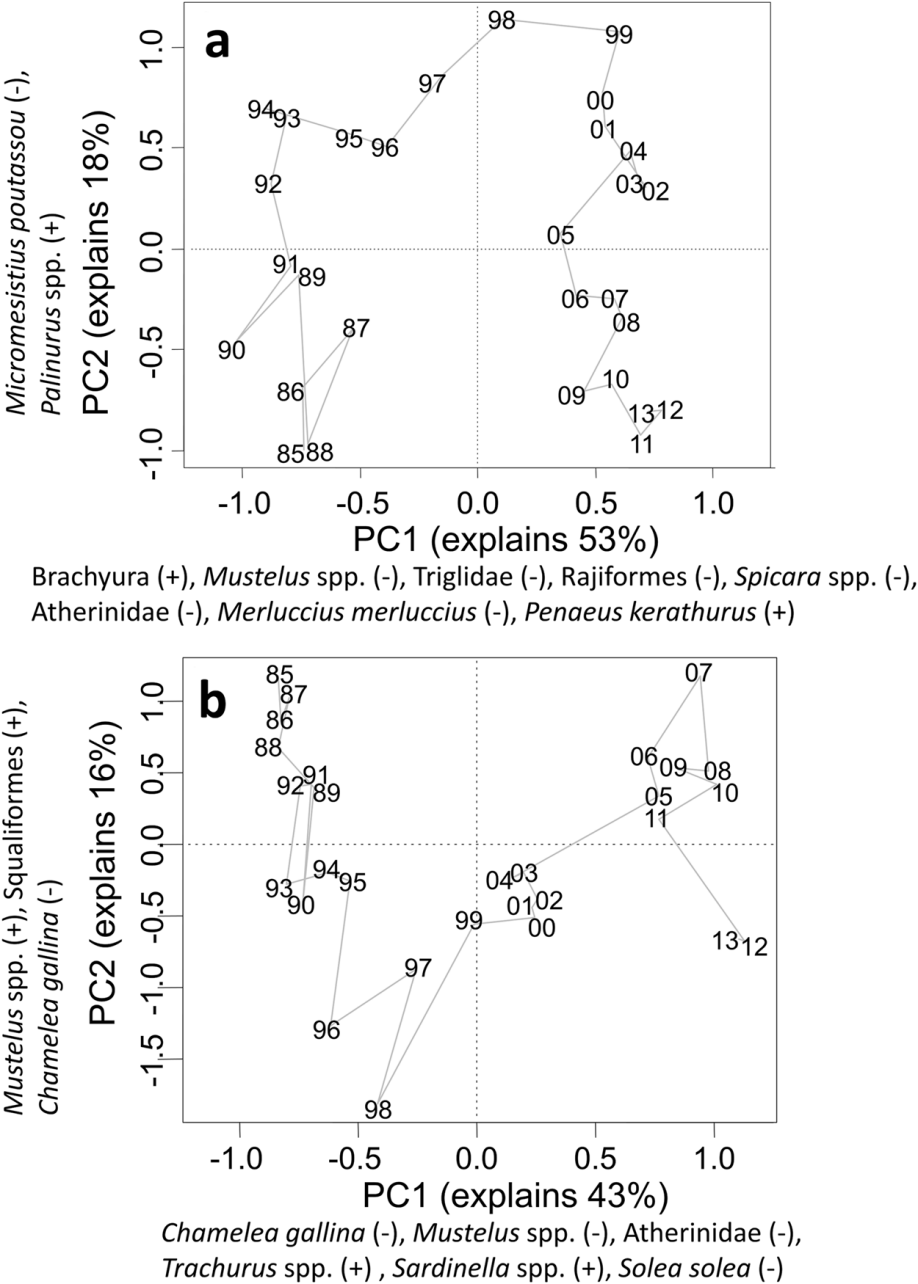
Yearly PC-scores along the first two principal components of the PCAs carried out for the eastern and the western Mediterranean systems. (**a**) The outputs of PCAeast carried out for the eastern Mediterranean system. (**b**) The outputs of PCAwest carried out for the western Mediterranean system. The positive (+) and negative (−) loadings of taxa with loadings of an absolute value higher than 0.5 on the first two principal components are displayed. The prefix 19- and 20- of the years is omitted.

The majority of the individual components of the eastern and western Mediterranean systems exhibited decreasing trends during 1985–2013 (Fig. 2). Notably, taxa with non-decreasing trends referred primarily to thermophilic organisms (preferred temperature of 18 °C or more). Two principal component analyses (PCAeast and PCAwest) were carried out to decompose the variability of the datasets into new composite variables (principal components; PCs) that would act as holistic system indicators (Table 1, Step 1). In the eastern Mediterranean system, plotting PC2east against PC1east indicated a transition along the x-axis during 1996–1999 (Fig. 3a), expressed as a step-change in PC1east (Supplementary Fig. S3a). In the western Mediterranean system, plotting PC2west against PC1west also indicated a transition along the x-axis during 1996-1999, and a second transition during 2004–2005 (Fig. 3b), which were expressed as two step-changes in PC1west (Supplementary Fig. S3b). These transitions in PC1east and PC1west were due to the contrasting loadings of different thermophilic and non-thermophilic taxa (Fig. 3; Supplementary Table S1).

PC1east and PC1west explained a high proportion of the total variability of their respective systems (53% and 43%, respectively) and captured the existence of alternate regimes; hence, they were retained as system indicators^9^. Preliminary investigation of the relationship between PC1east, PC1west and the temporal anomalies of their respective SSTs at 0- to 2-year lags, revealed statistically significant cross-correlations at the 0.05 level, after accounting for temporal autocorrelation (Supplementary Table S2).

### Non-additive modelling

To investigate the possibility of discontinuous system responses to sea warming (Table 1, Step 2), the fits of generalised additive models (GAMs) and non-additive threshold GAMs (TGAMs)^35^ on the relationships between -PC1east, -PC1west and their respective SSTs (SSTeast, SSTwest) at 0- to 2-year lags were compared (Table 1, Step 2). Negative values of PC1east and PC1west were used, because in fold bifurcations it is customary for older states to have higher y-values^3^. A TGAM with 1-year lagged SSTeast and a TGAM with 2-year lagged SSTwest as explanatory variables were found to provide the optimal fits in the eastern and western Mediterranean system, respectively (Table 2). This finding indicated the existence of discontinuous responses to sea warming in both systems. Years 1996 and 1998 were identified as threshold years (last years of the older regime) for the eastern and western Mediterranean system, respectively (Supplementary Fig. S4a,b). In the western Mediterranean system, an additional comparison between the fits of a GAM and a TGAM in years 1999–2013 was carried out, due to the erroneous residual distribution over the lower branch of the first TGAM (Supplementary Fig. S5b); thus, a second discontinuous response of the western Mediterranean system was unveiled in 2004 (Supplementary Fig. S4c). Ultimately, this analytical process revealed a fold-bifurcation with two alternate attractors in the eastern Mediterranean system, and two fold-bifurcations with three alternate attractors in the western Mediterranean system (Supplementary Fig. S5a,c).

**Table 2:** The ‘genuine’ CV values (gCV) and percentage of deviance explained (in brackets) of GAMs and TGAMs fitted on the relationships between PC1east, PC1west and SSTeast, SSTwest, respectively, at 0- to 2-year lags. Bold font indicates the models with the lowest gCV values (optimal models).

SST lag	Eastern Mediterranean		Western Mediterranean	
	GAM gCV	TGAM gCV	GAM gCV	TGAM gCV
0-lag	0.179 (67%)	0.0312 (97%)	0.339 (46%)	0.361 (86%)
1-lag	0.185 (68%)	**0.0297 (97%)**	0.336 (48%)	0.181 (88%)
2-lag	0.201 (59%)	0.0871 (93%)	0.305 (47%)	**0.124 (86%)**

### Resilience assessment

**Figure 4:**
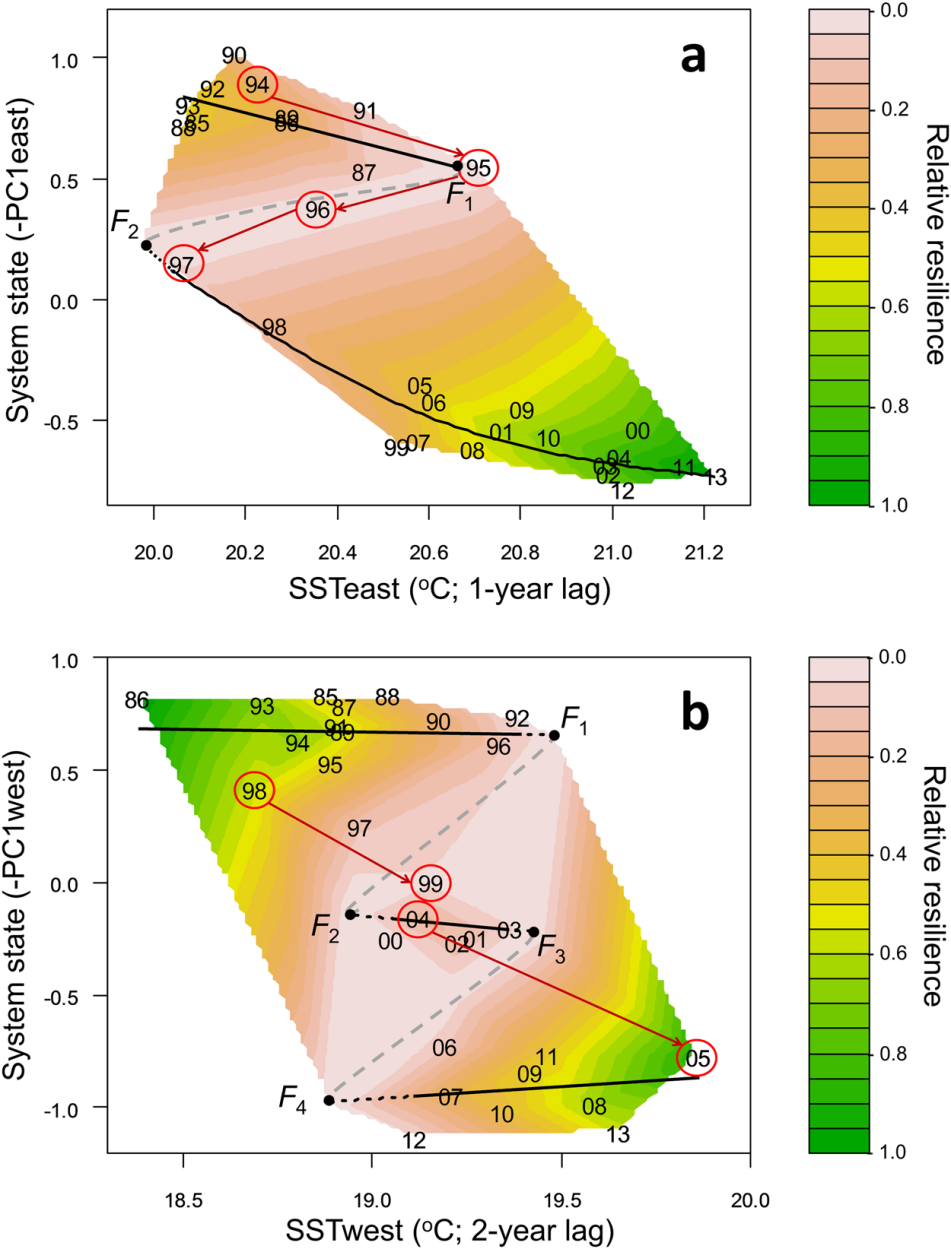
Empirical folded stability landscapes of the eastern and western Mediterranean systems. (**a**) The folded stability landscape of the eastern Mediterranean system exhibited two basins of attraction and two tipping points (*F*_1_, *F*_2_). (**b**) The folded stability landscape of the western Mediterranean system exhibited three basins of attraction and four tipping points (*F*_1_–*F*_4_). Continuous black lines indicate the attractors, dotted black lines indicate the possible extensions of the attractors, grey dashed lines indicate the borders of the basins of attraction, and red circles and arrows indicate the pathways of the regime shifts. Note that the x-axes refer to lagged SSTs. The prefix 19- and 20- of the years is omitted.

For the resilience assessment (Table 1, Step 3), system-specific relative resilience estimates were calculated for each year (*rRes*_y_) (Supplementary Fig. S6). Linear interpolation of the *rRes*_y_ estimates onto 100 × 100 grids led to the emergence of the folded stability landscapes of the eastern and western Mediterranean systems (Fig. 4). The folded stability landscape of the eastern Mediterranean system exhibited two basins of attraction (regimes); in 1994–1998, a shift commencing concurrently with an abrupt increase of SSTeast by 0.5 °C led the eastern Mediterranean system into a new basin of attraction (Fig. 4a). Years right after 1995 provided evidence for hysteresis, which is indicative of fold-bifurcations^3^: years 1996 and especially 1997–1998 corresponded to a similar range of SSTs with years 1985–1994, but exhibited a markedly different system configuration (y-axis values). The folded stability landscape of the western Mediterranean system exhibited three alternate basins of attraction (Fig. 4b). The relevant regime shifts in the western Mediterranean system were associated with distinct jumps in SSTwest of 0.5 and 0.7 °C for the first and second shift, respectively. The second basin of attraction in the western Mediterranean system (1999–2004) was much shallower (less resilient) than the first and third ones, implying that it possibly represented a transitional regime (Fig. 4b). Hysteresis effects were evident in both the eastern and western Mediterranean systems. These were illustrated by years of alternate regimes exhibiting different system configurations despite the substantial overlap in their SST values (Fig. 4).

## Discussion

In this study, the IRA framework (Table 1) was applied to two complex natural systems, revealing the occurrence of multiple fold-bifurcations, regime shifts, and alternate basins of attraction, shaped as predicted by theory^3,6,7^. This combination of multivariate analysis, non-additive modelling, and resilience assessment elucidated previously unknown system dynamics and shift attributes, and allowed to apply the concepts of resilience and folded stability landscapes in an empirical multivariate context. Our findings are consistent with previously identified effects of warming on Mediterranean ecosystems, such as shifts in the landings of specific taxa^32^, the increase in the mean temperature of the catch^4,30^, and changes in the biology, abundance and range of different species^31^. This study expands on previous findings by showing that the response mechanism of the exploited components of the Mediterranean biotas to sea warming is discontinuous, and that multiple abrupt regime shifts have occurred, which are possibly irreversible due to hysteresis^3,6^.

The timing of the regime shifts identified here was associated in all cases with pronounced increases in SST, in agreement to the dynamics expected in complex natural systems with multiple basins of attraction^3^. The importance of the 1993 and 2003 warming events for the development of Mediterranean biotas has also been identified in previous studies^31^, while the global acceleration in the rise of SST after the 1997/1998 El Niño event^36^ played an important role in the observed consolidation of the new regimes in the Mediterranean Sea. A possible stabilising mechanism of the alternate regimes detected here could be the differential species composition itself. The conditions triggering a new regime are not equally favourable for all species in the first place; species interactions are restructured according to the triggered new abundances and dynamics, and are affected by predation and competition, ultimately shaping a new stable state which acts as a basin of attraction. There is a need for further research to infer the exact biological mechanism(s) behind the way that SST year-to-year oscillations parallel the annual fluctuations of individual taxa, and the meaning of the time-lag of the SST effects. Approaches based on ecological niche theory^37,38^ and/or traits-based approaches^39,40^ could be used in that direction.

For the IRAs of this study, SST was used as the only stressor of the Mediterranean systems. Data availability for other important stressors, such as fishing pressure, primary production, and planktonic assemblages could have further elucidated the system dynamics. For example, the decreasing trends observed in the majority of the Mediterranean fisheries resources during 1985–2013, especially in thermophilic taxa of high economic value such as *Merluccius merluccius* and *Epinephelus* spp., and in vulnerable taxa such as the elasmobranchs, possibly also reflect the overfishing problem in the Mediterranean Sea^41,42^. Overfishing could also be partly responsible for the decrease in commercially important, non-thermophilic taxa, such as *Solea solea* and *Palinurus* spp., but in that case it is harder to disentangle the effects of fishing and sea warming. Unfortunately, datasets of additional system stressors were not available over the wide spatial and temporal scale of this study. Intriguingly, even in such a highly complex biodiversity hotspot exposed to many different anthropogenic and natural stressors^29^, the effect of sea warming is discernible, illustrating the pivotal role of temperature in the ecosystem configuration of the Mediterranean Sea.

The FAO landings data which were used to demonstrate the application of the IRA framework in an empirical context are known to include a certain degree of uncertainty^43,44^. In any case, landings data are expected to provide a generally adequate picture of the trends of different exploited taxa if conditioned properly^45,46^, which was the case in this study (Supplementary Methods). The FAO GFCM landings dataset has been frequently analysed in studies of the development of marine ecosystems in the Mediterranean Sea^4,30,32,47^. Also, recent studies estimating reconstructed catches (FAO landings plus discards and unreported catches) in the Mediterranean Sea show that the trends of total FAO landings and reconstructed catches are very similar^48,49^. Even if fisheries landings may sometimes lead to misleading conclusions regarding stock status, the fact that abrupt multivariate shifts in Mediterranean fisheries landings were detected in a period without abrupt changes in fishing effort^32^ supports the hypothesis of climate-induced regime shifts in the Mediterranean Sea.

This is a study that presents a new methodology which is applied to a fisheries landings dataset. To further support our findings regarding the impact of temperature on the Mediterranean marine communities, additional analyses should be developed using alternative datasets. Unfortunately, there is absence of other long-term time-series for Mediterranean fisheries resources covering the entire basin. Time-series from other sources, such as surveys^50^ or stock assessments^51^, are available over much shorter time periods, cover only the EU seas, and refer to stocks of relatively small sub-areas. For example, the Mediterranean International Bottom Trawl Survey (MEDITS)^50^, which is the longest-running comprehensive survey of fisheries resources in the Mediterranean, commenced in the mid-1990s in some areas and after 2000 in others, covers only the northern part of the basin, and targets just demersal species. Unlike FAO data, MEDITS data are not publicly available, and a pan-European effort would be needed to collect, aggregate and homogenise these data for use in a multivariate analysis. Nevertheless, such an analysis of MEDITS data would be worthwhile and should be attempted in the future as it would produce valuable results, albeit at a different spatial, temporal and biological scale than our study.

We envisage that our findings will contribute towards an Ecosystem Approach to Fisheries Management in the Mediterranean Sea, and promote the incorporation of the resilience perspective into the regional management guidelines^52^. For example, stock assessments and advice need to be tailored to the latest ecosystem regimes and resilience, and predictions of future stock dynamics should take into account the possible discontinuous effects of a further sea warming^53^ on the Mediterranean marine communities.

It should be noted that the IRA framework is not a panacea, and stability landscapes cannot be constructed for all kinds of system dynamics. If the comparison between a continuous and a discontinuous system-stressor relationship (Supplementary Fig. S1) is in favour of the former (Table 1, Step 2), i.e. if a GAM exhibits a better fit than a TGAM, then it can naturally be deducted that there are no fold-bifurcations or alternate basins of attraction. Even if the occurrence of a discontinuous system response is established, the construction of a stability landscape requires regime shifts to coincide with sensible changes in stressors, such as the SST jumps observed in the current study. If a system exhibits regime shifts when the stressors are not changing, or when stressors change contrary to the underlying biological mechanisms, there are probably other stressors at play driving the system dynamics.

The analytical framework introduced here is transferable to other shifting marine or terrestrial complex natural systems at different levels of biological organization. It can also be used in the study of social, economic^3,15^ or behavioural^54^ complex systems exhibiting non-linear responses to changing stressors. Meanwhile, there are several empirical natural systems with available data series describing the development of multiple system components and stressors, and in some of them regime shifts have already been detected^3,9^. Applying the IRA framework to such empirical systems in order to assess response types, quantify resilience dynamics, and construct folded stability landscapes would greatly enhance our understanding of system dynamics and shift mechanisms. This could support environmental sustainability and human welfare in a fast-changing world.

## Methods

### Landings dataset

The FAO GFCM fisheries landings data were extracted from the FAO database using the FishSTATJ software^55^. Landings data were available for the period 1970 to 2013, but only data from 1985 to 2013 were used, due to the lower data quality before 1985^32^. Of the 30 taxa compiled and analysed; 11 taxa referred to the species level, nine taxa referred to the genus level, five taxa referred to the family level and five taxa included species from more than one family. Full details on the construction of this dataset are available in the Supplementary Methods.

### Preferred temperatures dataset

The median preferred temperatures for each of the analysed taxa were extracted from the relevant dataset produced by^4^. These preferred temperatures refer to the SST of the areas where species are distributed, and not to the ambient temperature^4^. Full details on the construction of this dataset are available in the Supplementary Methods.

### SST dataset

The Level 4 mapped, gap-free, blended AVHRR (Advanced Very High Resolution Radiometer, *Pathfinder v5*) SST product was acquired from the NASA PO.DAAC^56^. The data are corrected with *in situ* observations (acquired from buoys and ships), and mapped on a 0.25° × 0.25° resolution grid using optimal interpolation^57^. Monthly SST (merged day and night) datasets were obtained from podaac.jpl.nasa.gov for the period 1982 to 2015. The monthly aggregates were spatially averaged over the eastern and western Mediterranean Sea, and they were also temporally averaged to produce the final SST dataset with annual resolution (Fig. 1b). The accuracy of the AVHRR Pathfinder SST estimates is documented in several studies^58,59^. The Pathfinder SST is a dependable dataset for studying global and regional trends and anomalies^58,60^.

### IRA step 1: Complexity reduction

To investigate the multivariate temporal development of the eastern and western Mediterranean systems and reduce their complexity (Table 1, Step 1), two separate PCAs were carried out on the 30 variables (taxa) that were compiled. The PCAs were based on the covariance matrices of the variables, following log-transformation of all variables to ensure normal distributions. PCAs are frequently used in integrated assessments, as they convert sets of possibly correlated variables into new linearly uncorrelated composite variables (PCs) using an orthogonal transformation of the data to a new coordinate system^9–11^. To identify the key taxa driving the system trends through time, the contributions of all original taxa on the PC-scores (loadings) along the first and second PCs (PC1 and PC2) were calculated for both PCAeast and PCAwest^9^. Examination of the loadings of different taxa on PC1east, suggested that the observed transition was mainly due to the increase of Brachyura and *Penaeus kerathurus* (high positive loadings), and the decrease of *Mustelus* spp., Triglidae, Rajiformes, *Spicara* spp. and Atherinidae (high negative loadings) (Supplementary Table S1). The transitions of PC1west were mainly due to the decrease of *Chamelea gallina*, *Mustelus* spp., Atherinidae and *Solea solea* (high negative loadings) and the increase of *Trachurus* spp. and *Sardinella* spp. (high positive loadings) (Supplementary Table S1). The structure of all taxa was visualized using the traffic light framework^9–11^. For this, taxa were sorted according to their loadings on PC1, logged values of each taxon were categorized into quintiles, and each quintile was given a specific colour ranging from green (lowest quintile) to red (highest quintile).

The multivariate temporal development of the eastern and western Mediterranean systems in 1985–2013 was visualized by plotting the PC-scores of each year along the PC1 and PC2 axes on a two-dimensional coordinate system, separately for PCAeast and PCAwest. PC1east and PC1west were used as holistic indicators of their respective systems as they captured a substantial proportion of the total variability of the datasets^9^. The sequential regime shift detection method (STARS), modified to account for autocorrelation by adjusting the degrees of freedom of the regime shift index (RSI)^61^, was applied to detect significant (*p* < 0.05) shifts in the mean values of PC1east and PC1west in 1985–2013.

For a preliminary investigation of the SST effect prior to the non-additive modelling, the temporal development of the system indicators PC1east and PC1west in 1985–2013 was plotted against their respective SST anomalies, and Pearson cross-correlation (r) analysis was implemented to examine the relationships between the system indicators and SST at 0- to 2-year lags. The probability of significance of the correlation was adjusted to correct for temporal autocorrelation (*P*_ACF_) using the Chelton method, which calculates an ‘effective’ number of degrees of freedom, penalizing for autocorrelation found at five different time-lags^62^.

Note that the visualisation of PC-scores, the application of STARS, the cross-correlation analysis, and the examination of preferred temperatures were not essential for the IRA, but they assisted in a better understanding of the system dynamics.

### IRA step 2: Non-additive modelling

To investigate the possibility of discontinuous relationships between the system indicators and SST (Table 1, Step 2), -PC1east and -PC1west were used as response variables and their respective SSTs with 0- to 2-year lags were used as explanatory variables in GAMs and TGAMs. Negative values of PC1east and PC1west were used, because it is customary in fold-bifurcations for chronologically older alternate states to have higher y-values^3^. GAMs assume additive and stationary relationships between the response and explanatory variables, while TGAMs can represent an abrupt change in the relationships between the response and explanatory variables (i.e., a threshold) in a specific year^8,35^. To compare the fit of continuous (GAMs) and discontinuous models (TGAMs) on the relationships between the system indicators -PC1east, -PC1west and their respective SSTs at different time lags, the ‘genuine’ cross-validatory squared prediction error (gCV) was computed^35^. The gCV accounts for the estimation of the threshold line and the estimation of the degrees of freedom for the functions appearing in all additive and non-additive formulations. In TGAMs, the threshold years were selected by minimizing the GCV of the whole model through the implementation of a searching algorithm which runs the model for 53 possible threshold years between the 0.1 lower and the 0.9 upper quantiles^8^.

TGAMs can only detect a single threshold in the relationships between the response and explanatory variables. However, the TGAM branch fitted over years 1999–2013 in the western Mediterranean system produced an erroneous residual distribution, with years 1999–2004 and 2005–2013 forming two distinct clusters (Supplementary Fig. S5b). This hinted at the existence of two regimes in 1999–2013; hence, the fits of a GAM and a TGAM on the relationship between -PC1west and 2-year lagged SSTwest in 1999–2013 were also compared. The fitted TGAM (gCV = 0.130) was found to provide a better fit than its respective GAM (gCV = 0.158), indicating a second discontinuous response in the western Mediterranean system in 1999–2013. The threshold year that minimized the GCV value of the TGAM fitted over years 1999–2013 was 2005 (Supplementary Fig. S4c). However, a Chi-squared test suggested that the difference between the residual deviance of TGAMs with 2005 or 2004 as threshold years was non-significant (*p* = 0.148), and that a TGAM with 2004 as threshold year was more parsimonious. For this, a TGAM with 2004 as threshold year was retained as the optimal model for the western Mediterranean system for the period 1999–2013 (Supplementary Fig. S5c).

### IRA step 3: Resilience assessment

To estimate annual resilience values (*Res*_y_) (Table 1, Step 3), the sum of the horizontal distance of each year from the tipping point of its regime (horizontal resilience component; *hComp*_y_) minus the vertical distance of each year from its fitted attractor (vertical resilience component; *vComp*_y_) was used. This calculation requires the x- and y-axis to be measured at the same scale^27^; hence, the mean-standardised values of SST were used for the calculation of *hComp*_y_, as PC1 scores are also mean-standardised. To calculate the position of the tipping point(s) of each regime along the trajectory defined by the regime’s attractor, the x-coordinate of the tipping point was rounded to the nearest 0.05 °C allowing all *Res*_y_ within each regime to be positive. It should be noted that the positions of tipping points in empirical systems cannot be known exactly because these points are not empirical observations. This is a common issue with tipping points in empirical systems^3,7^. However, the uncertainty regarding the exact position of the tipping points is not particularly important here, because a somewhat different placement of the tipping points along the attractor trajectories, with the restriction of ensuring positive *Res*_y_ estimates within each regime, would not affect the general shape of the basins of attraction or the observed shift mechanisms^27^.

In the eastern Mediterranean, year 1995 was associated with a pronounced SSTeast increase and years 1996–1998 corresponded to much different states than years 1985–1994 despite being under the influence of a similar SSTeast (Fig. 4a); hence, *F*_1_ was expected to lie along the upper branch of the fitted TGAM, between the 1985–1994 year-cluster and year 1995. Therefore, transitional years 1995 and 1996 were attributed zero *Res*_y_ and tipping point *F*_1_ was assumed to lie 0.05 °C below the SSTeast in 1995. Tipping point *F*_2_ was assumed to lie at the lowest SSTeast value (rounded to the nearest 0.05 °C) ensuring that all *rRes*_y_ estimates of the new regime would be non-negative. Similarly to the eastern Mediterranean system, the tipping points of the western Mediterranean system (*F*_1_–*F*_4_) were assumed to lie at the nearest SSTeast value of their respective year-clusters (rounded to the nearest 0.05 ^o^C) resulting in all *Res*_y_ estimates of each regime to be positive (Fig. 4b).

*Res*_y_ was scaled by dividing with the maximum value observed in each system to calculate relative resilience (*rRes*_y_). An *rRes* value of zero was attributed to all tipping points and transitional years, and a theoretical point of zero *rRes* was added at (19.2, −0.5) of the western Mediterranean folded stability landscape to facilitate the correct calculation of the lower basin border. Linear interpolation of all *rRes*_y_ values onto a 100 × 100 grid, separately for the eastern and western Mediterranean systems created two filled contour plots representing the three-dimensional folded stability landscapes. On these folded stability landscapes, areas of high or low system resilience within different regimes could be linked to specific SST levels, as predicted by theory^3,6,7^.

### Data availability

All data and R scripts are available upon request to the corresponding author.

## Electronic supplementary material

Supplementary Information
