# Supplementary material for: Resilience and regime shifts in a marine biodiversity hotspot
Source: Sci Rep. 2017 Oct 20;7:13647. doi: 10.1038/s41598-017-13852-9 (PMC5651905; doi:10.1038/s41598-017-13852-9)
Supplement: Supplementary file 1 — Supplementary Information [file 41598_2017_13852_MOESM1_ESM.pdf]

Supplementary Information

Resilience and regime shifts in a marine biodiversity hotspot

Paraskevas Vasilakopoulos; Dionysios E. Raitsos; Evangelos Tzanatos; Christos D. Maravelias

Supplementary Methods

Compilation of the landings dataset. FAO landings data from the Mediterranean Sea were available for the period 1970 to 2013, but only data from 1985 to 2013 were used, due to the lower data quality before 1985[1]. Landings from the Mediterranean FAO sub-areas 1.1 (Balearic), 1.2 (Gulf of Lions) and 1.3 (Sardinia) were grouped together into the western Mediterranean landings dataset, while landings from sub-areas 2.1 (Adriatic), 2.2 (Ionian), 3.1 (Aegean) and 3.2 (Levantine) were grouped together into the eastern Mediterranean landings dataset (Fig. 1a). This division was due to the different ecological characteristics[2] and warming profiles[3] of the eastern and western Mediterranean sub-areas. It also ensured the availability of a large number of taxa present in both areas to allow the implementation of informative and comparable IRAs. A division into smaller groups of sub-areas or at a sub-area level was avoided because it would result in fewer taxa being present in all areas examined.

Landings from the western and eastern Mediterranean were divided into groups (taxa) with the aim of creating groups at the lowest possible taxon (species level). Care was taken to have the same taxa present in both the western and the eastern Mediterranean datasets. For example, alien species from tropical seas were excluded from the analysis as they are caught in large quantities mainly in the eastern Mediterranean[4]. Inconsistencies in the FAO landings time-series, such as the extirpation of the landings of a species in certain years and concurrent

increase of the landings of its genus, or greater landings attributed to a genus or family compared to the landings of individual species belonging to that genus or family, were treated by grouping to the higher taxon hierarchically. Important Mediterranean aquaculture production species (e.g. *Sparus aurata*, *Dicentrarchus labrax, Pagrus pagrus, Mytilus* spp. etc.) were excluded from the analysis, because the natural abundance and the subsequent catches of these species could be affected by aquaculture escapees. Highly migratory large pelagic fishes (e.g. *Thunnus* spp., *Xiphias gladius* etc.) were also excluded, because of the possibility of migrations between the Atlantic and the Mediterranean, and the existence of catch quotas for *Thunnus thynnus*.

The 30 taxa that were compiled and analysed in the eastern and western Mediterranean Sea were as following:

Bony fishes:

1. *Engraulis encrasicolus*
2. *Sardina pilchardus*
3. *Micromesistius poutassou*
4. *Merluccius merluccius*
5. *Boops boops*
6. *Solea solea*
7. *Lophius* spp.
8. *Sardinella* spp. (includes FAO categories *Sardinella* spp. and *Sardinella aurita*)
9. *Trachurus* spp. (includes FAO categories *Trachurus* spp., *Trachurus mediterraneus* and *Trachurus trachurus*)
10. *Scomber* spp. (includes FAO categories *Scomber* spp., *Scomber scombrus* and *Scomber japonicus*)

11. *Spicara* spp. (includes FAO categories *Spicara* spp., *Spicara maena* and *Spicara smaris*)
12. *Epinephelus* spp. (includes FAO categories *Epinephelus* spp., *Epinephelus marginatus, Epinephelus aeneus* and *Epinephelus caninus*)
13. Mugilidae (includes FAO categories Mugilidae, *Mugil cephalus, Liza aurata, Chelon labrosus*)
14. *Mullus* spp. (includes FAO categories *Mullus* spp., *Mullus barbatus, Mullus surmuletus*)
15. Triglidae (includes FAO categories Triglidae, *Eutrigla gurnardus*, *Chelidonichthys lucerna* and *Aspitrigla cuculus*)
16. Atherinidae (includes FAO categories Atherinidae, *Atherina boyeri* and *Atherina hepsetus*)
17. Scophthalmidae (includes FAO categories Scophthalmidae, *Scophthalmus rhombus Lepidorhombus whiffiagonis* and *Psetta maxima*)

Chondricthyan fishes:

18. *Mustelus* spp. (includes FAO categories *Mustelus* spp. and *Mustelus mustelus*
19. Rajiformes (includes FAO categories Rajiformes, Rhinobatidae, *Raja* spp., *Raja alba, Raja clavata*, *Raja naevus*, *Raja montagui*, and *Rhinobatos rhinobatos*)
20. Squaliformes (includes FAO categories Squalidae, *Squalus* spp., *Squalus acanthias, Squalus blainville*, *Etmopterus spinax*, *Centroscymnus coelolepis* and *Centrophorus granulosus*).

Crustaceans:

21. *Squilla mantis*
22. *Penaeus kerathurus*
23. *Nephrops norvegicus*
24. *Parapenaeus longirostris*

25. *Palinurus* spp. (includes FAO categories *Palinurus* spp., *Palinurus elephas* and *Palinurus mauritanicus*)
26. Brachyura (includes FAO categories Brachyura, *Geryon longipes*, *Carcinus aestuarii* and *Maja squinado*)

Molluscs:

27. *Chamelea gallina*
28. Octopodidae (includes FAO categories Octopodidae, *Octopus vulgaris* and Eledone spp.)
29. Sepiidae-Sepiolidae (includes FAO categories Sepiidae-Sepiolidae and *Sepia officinalis*)
30. Loliginidae-Ommastrephidae (includes FAO categories Loliginidae-Ommastrephidae, *Loligo* spp., *Illex coindetii* and *Todarodes sagittatus*)

Some obvious mistakes in the FAO landings database were corrected during the construction of the datasets. In the western Mediterranean dataset, for Squaliformes (cat. 20), the average value of landings of years 1984 and 1989 were used in years 1985-1988 when relevant landings were missing from the database. For Brachyura (cat. 30), the average value of landings of years 1989 and 1996 were used in the Balearic area (1.1) in years 1990-1995 when relevant landings were also missing from the database. In the eastern Mediterranean dataset, the Squaliformes landings reported by Libya from the Ionian area (2.2) in 2009-2013 were omitted from the total Squaliformes landings (cat. 20) as erroneous, because they were almost 10 times higher than Squaliformes landings from all the other Mediterranean countries put together, and followed 30 years of no Libyan Squaliformes landings.

Calculation of preferred temperatures. The species-specific median preferred temperature[5] was used for the 11 taxa that referred to a single species. In seven more taxa which referred to a higher than species level, landings were dominated by a single species and the median

preferred temperature of that species was attributed to the taxon. In two more taxa where landings were dominated by two species with similar median preferred temperatures (difference of 1-2 ^{o}C), the average median preferred temperature of these two species was attributed to the taxon. No preferred temperatures were attributed to the 10 remaining taxa which included species with greater than 2 ^{o}C difference in preferred temperature, and with similar or unknown relative contribution to the taxon's landings.

Specifically, for the single-species taxa *Engraulis encrasicolus*, *Sardina pilchardus*, *Micromesistius poutassou*, *Merluccius merluccius*, *Boops boops*, *Solea solea*, *Squilla mantis*, *Penaeus kerathurus*, *Nephrops norvegicus*, *Parapenaeus longirostris* and *Chamelea gallina* the species-specific preferred temperature was used. For taxon *Lophius* spp., the preferred temperature of *Lophius budegassa* was used; for taxon *Sardinella* spp., the preferred temperature of *Sardinella aurita* was used; for taxon *Epinephelus* spp., the preferred temperature of *Epinephelus marginatus* was used; for taxon Atherinidae, the preferred temperature of *Atherina boyeri* was used; for taxon *Mustelus* spp., the preferred temperature of *Mustelus mustelus* was used; for taxon *Palinurus* spp., the preferred temperature of *Palinurus elephas* was used; for taxon Sepiidae-Sepiolidae, the preferred temperature of *Sepia officinalis* was used. For taxon *Trachurus* spp., the mean preferred temperature of *Trachurus trachurus* and *Trachurus mediterraneus* was used, and for taxon *Mullus* spp., the mean preferred temperature of *Mullus surmuletus* and *Mullus barbatus* was used.

Supplementary Figures

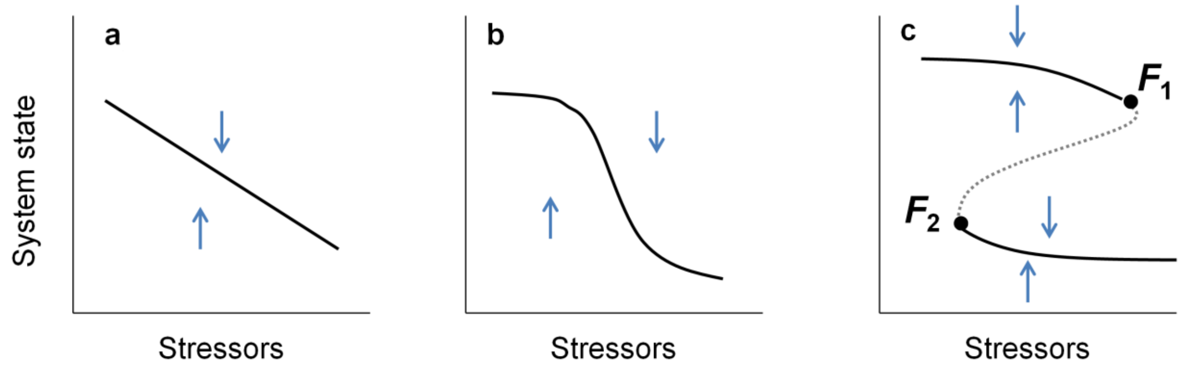

Supplementary Figure S1. Possible ways that a complex natural system can respond to changing stressors such as temperature, exploitation, nutrients etc. System response can be continuous linear (**a**) or sigmoid (**b**), but it can also be discontinuous, where the response curve is folded backwards at the tipping points F_1 and F_2 (**c**). Arrows indicate the attraction forces towards the response curve (mean state). Adjusted from [6].

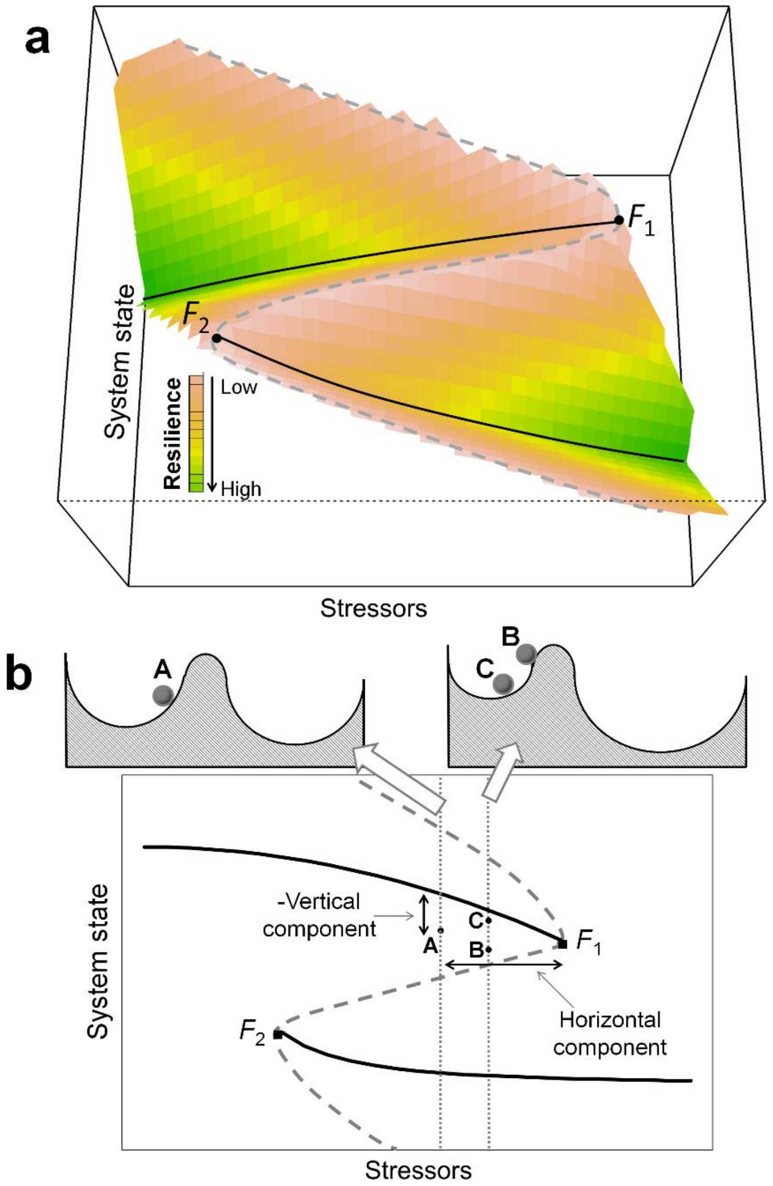

Supplementary Figure S2. A generic folded stability landscape with two basins of attraction. System response curves (attractors) and basins' borders are indicated by black continuous and grey dashed lines, respectively. As conditions change and the system approaches a tipping point (F_1 or F_2) resilience erodes and the basins of attraction become narrower and shallower (**a**). The horizontal distance of a state from the tipping point expresses its horizontal component of resilience (hComp), while the distance of a state from its attractor curve expresses its negative vertical component of resilience (-vComp) (**b**). The negative sign indicates that the greater the distance of a state from its attractor, the lower its resilience. Resilience of state A can be calculated as ResA = hCompA + vCompA. States B and C have the same hComp but different vComp; hence, state C is more resilient than state B. States A and B have the same vComp but different hComp; hence, state A is more resilient than state B. Adjusted from [7].

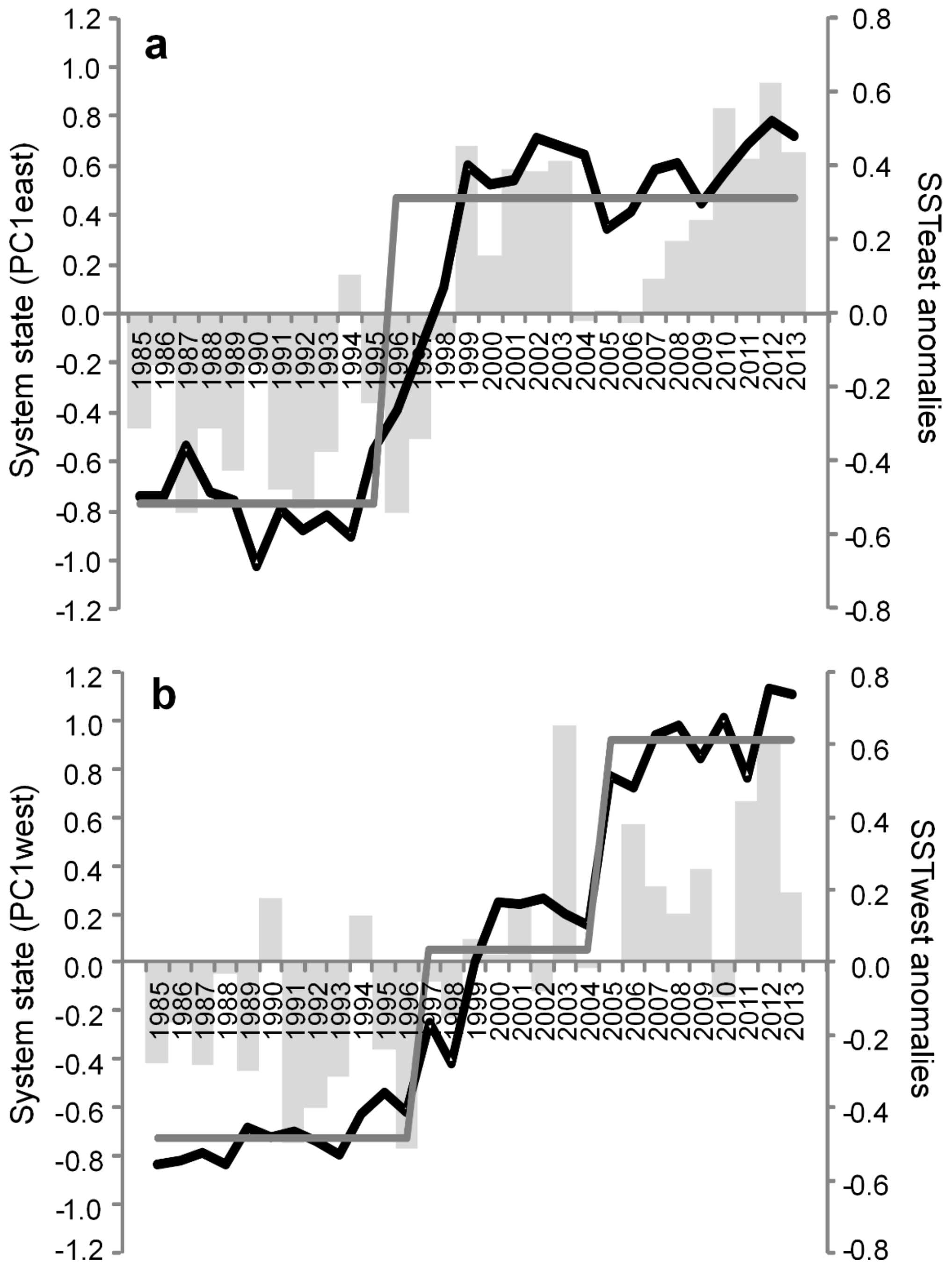

Supplementary Figure S3. The temporal development of the system indicators (first PCs) and SST anomalies in the eastern and western Mediterranean systems. The eastern Mediterranean system (**a**) exhibited a single step-change associated with increasing SSTeast. The western Mediterranean system (**b**) exhibited two step-changes associated with increasing SSTwest. Black lines indicate the temporal development of system state, grey lines indicate the step-changes in mean PC1east and PCwest values detected by STARS ($p < 0.05$), and grey histograms indicate the SST anomalies.

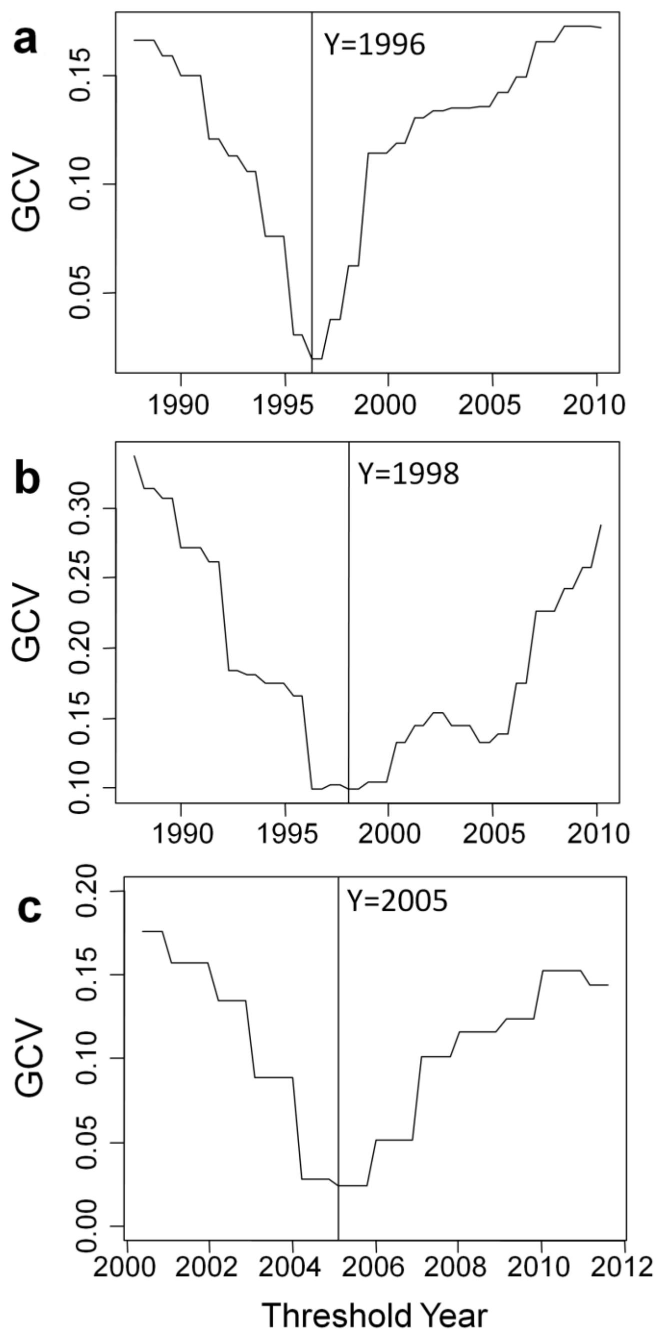

Supplementary Figure S4. GCV values of TGAMs with different threshold years fitted on the relationship between system state and SST. GCV of TGAMs fitted on the relationship between -PC1east and 1-year lagged SSTeast in 1985-2013 (**a**), on the relationship between -PC1west and 2-year lagged SSTwest in 1985-2013 (**b**), and on the relationship between -PC1west and 2-year lagged SSTwest in 1999-2013 (**c**). Upper and lower 10% of possible threshold years were omitted when applying the searching algorithm. Vertical lines indicate the threshold year resulting in the lowest GCV value in each case.

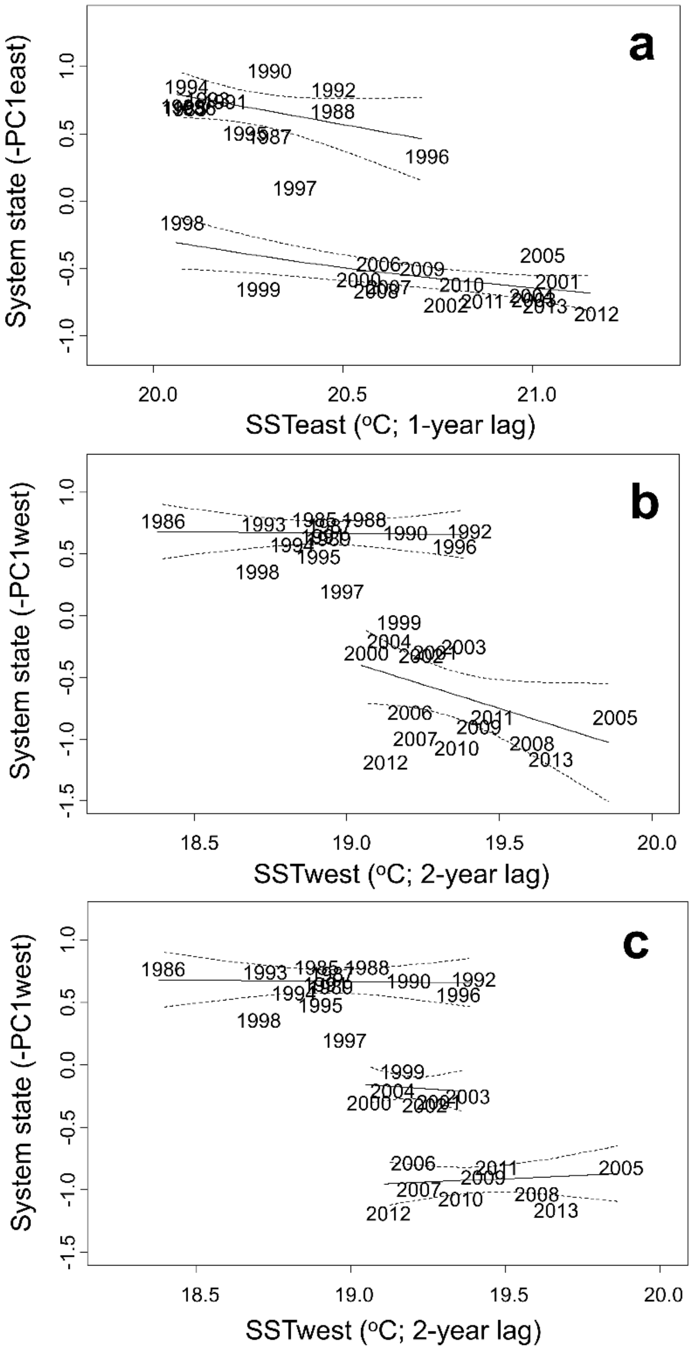

Supplementary Figure S5. The optimal TGAMs fitted on the relationships between the system state indicators and SST for the eastern and western Mediterranean systems. (**a**) The optimal TGAM for the relationship between -PC1east and 1-year lagged SSTeast. (**b**) The optimal TGAM for the relationship between -PC1west and 2-year lagged SSTwest with one threshold year. Note the erroneous distribution of residuals around the lower branch. (**c**) The TGAM for the relationship between -PC1west and 2-year lagged SSTwest with two threshold years which was found to provide a better fit than (**b**) hence it was retained as the optimal TGAM for the western Mediterranean system. Continuous lines indicate the fitted TGAMs and dashed lines indicate the confidence intervals.

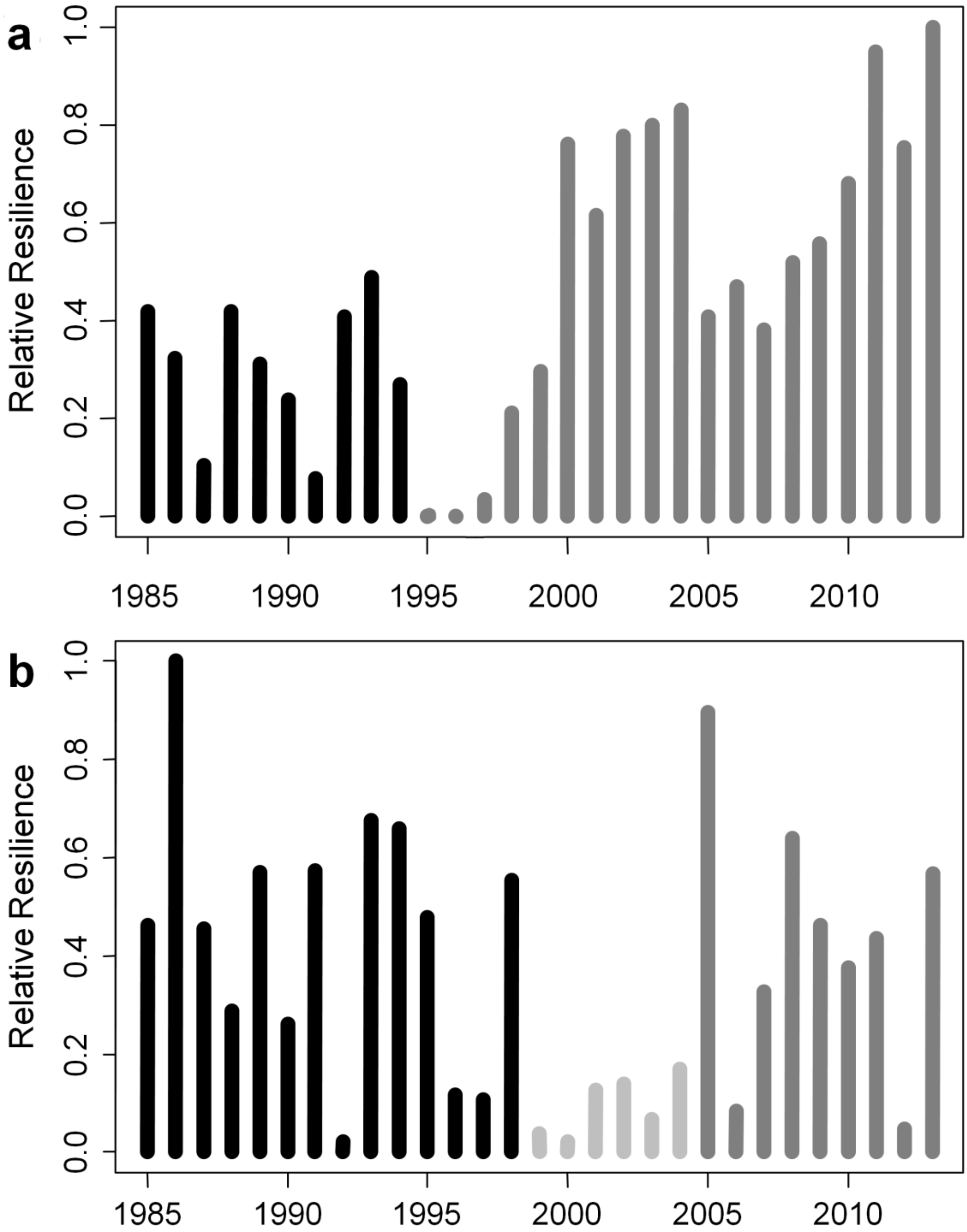

Supplementary Figure S6. Resilience assessment of the eastern and western Mediterranean systems during 1985-2013. (**a**) Resilience assessment of the eastern Mediterranean system exhibiting two regimes. (**b**) Resilience assessment of the western Mediterranean system exhibiting three regimes. Vertical lines indicate the relative resilience of each year and different colour shading indicates years of different regimes.

Supplementary Tables

Supplementary Table S1. Loadings of all taxa on the first two principal components of PCAeast and PCAwest. High positive or negative loadings (absolute values greater than 0.5) are shown in bold.

	Eastern Mediterranean		Western Mediterranean	
Taxa	PC1east	PC2east	PC1west	PC2west
Atherinidae	**-0.57**	0.40	**-0.83**	0.22
Boops boops	-0.22	0.04	0.31	0.08
Brachyura	**1.00**	-0.11	-0.12	0.19
Chamelea gallina	-0.10	0.16	**-1.33**	**-0.60**
Engraulis encrasicolus	0.47	0.00	-0.23	-0.04
Epinephelus spp.	-0.40	0.26	-0.44	0.27
Loliginidae-Ommastrephidae	-0.50	-0.19	-0.02	0.07
Lophius spp.	-0.21	-0.02	0.34	0.44
Merluccius merluccius	**-0.57**	-0.01	-0.12	0.13
Micromesistius poutassou	-0.45	**0.78**	-0.41	0.35
Mugilidae	0.04	0.33	-0.49	0.15
Mullus spp.	-0.03	0.01	0.00	0.18
Mustelus spp.	-0.98	-0.23	**-0.96**	**0.60**
Nephrops norvegicus	-0.45	-0.06	-0.25	0.12
Octopodidae	-0.29	0.00	0.08	0.10
Palinurus spp.	-0.50	**-0.70**	-0.40	0.19
Parapenaeus longirostris	-0.30	-0.43	-0.10	0.02
Penaeus kerathurus	**0.56**	0.03	0.45	-0.03
Rajiformes	**-0.64**	0.08	0.03	0.12
Sardina pilchardus	-0.26	-0.16	-0.25	0.09
Sardinella spp.	0.42	0.22	**0.56**	-0.37
Scomber spp.	-0.27	0.39	0.11	0.12
Scophthalmidae	-0.41	-0.19	-0.41	0.18
Sepiidae-Sepiolidae	-0.06	-0.11	-0.08	0.11
Solea solea	-0.30	-0.16	**-0.55**	0.14
Spicara spp.	**-0.63**	0.21	0.22	0.18
Squaliformes	-0.20	0.27	0.41	**0.80**
Squilla mantis	0.29	0.02	0.49	0.20
Trachurus spp.	-0.23	0.02	**0.60**	0.28
Triglidae	**-0.67**	-0.04	-0.05	0.01

Supplementary Table S2. The cross correlation coefficient (*r*) of PC1 and SST at 0- to 2-year lags for the eastern and western Mediterranean systems, and the penalised probability of non-significance of the correlation (P_{ACF}) after accounting for the temporal autocorrelation at five time-lags.

	Eastern Mediterranean		Western Mediterranean	
SST lag	*r*	P_{ACF}	*r*	P_{ACF}
No lag	0.82	0.012	0.68	0.022
1-year lag	0.77	0.021	0.69	0.014
2-year lag	0.76	0.022	0.68	0.022

Supplementary references

1. Tzanatos, E., Raitsos, D.E., Triantafyllou, G., Somarakis, S. & Tsonis A.A. Indications of a climate effect on Mediterranean fisheries. *Clim. Change* **122**, 41-54 (2014).
2. Garibaldi, L. & Caddy, J.F. Biogeographic characterization of Mediterranean and Black Seas faunal provinces using GIS procedures. *Ocean Coast. Manage.* **39**, 211-227 (1998).
3. Raitsos, D.E. *et al.* Global climate change amplifies the entry of tropical species into the eastern Mediterranean Sea. *Limn. Oceanogr.* **55**, 1478-1484 (2010).
4. Zenetos, A. *et al.* Alien species in the Mediterranean Sea by 2010. A contribution to the application of European Union's Marine Strategy Framework Directive (MSFD). Part I. Spatial distribution. *Med. Mar. Sci.* **11**, 381-493 (2010).
5. Cheung, W.W.L., Watson, R. & Pauly, D. Signature of ocean warming in global fisheries catch. *Nature* **497**, 365-368 (2013).
6. Scheffer, M., Carpenter, S., Foley, J.A. & Folke, C. Walker, Catastrophic shifts in ecosystems. *Nature* **413**, 591-596 (2001).
7. Vasilakopoulos, P. & Marshall, C.T. Resilience and tipping points of an exploited fish population over six decades. *Glob. Change Biol.* **21**, 1834-1847 (2015).
